# Cohorts as collections of bodies and communities of persons: insights from the SEARCH010/RV254 research cohort

**DOI:** 10.1093/inthealth/ihaa060

**Published:** 2020-11-09

**Authors:** Gail E Henderson, Stuart Rennie, Amy Corneli, Holly L Peay

**Affiliations:** Department of Social Medicine, University of North Carolina School of Medicine, 347A MacNider, 333 South Columbia Street, Chapel Hill, NC 27599-7240, USA; Department of Social Medicine, University of North Carolina School of Medicine, 347A MacNider, 333 South Columbia Street, Chapel Hill, NC 27599-7240, USA; Departments of Population Health Sciences and Medicine, School of Medicine, Duke Clinical Research Institute, Duke University, 215 Morris Street, Durham, NC 27701; RTI International, 3040 E Cornwallis Rd, Research Triangle Park, NC 27709

**Keywords:** cohort studies, cure trials, ethics, HIV, informed consent, social sciences

## Abstract

Longitudinal research cohorts are uniquely suited to answer research questions about morbidity and mortality. Cohorts may be comprised of individuals identified by specific conditions or other shared traits. We argue that research cohorts are more than simply aggregations of individuals and their associated data to meet research objectives. They are social communities comprised of members, investigators and organizations whose own interests, identities and cultures interact and evolve over time. The literature describes a range of scientific and ethical challenges and opportunities associated with cohorts. To advance these deliberations, we report examples from the literature and our own research on the Thai SEARCH010/RV254 cohort, comprising individuals diagnosed with human immunodeficiency virus (HIV) during acute infection. We reflect on the impact of cohort experiences and identity, and specifically how people incorporate cohort participation into meaning making associated with their diagnosis, the influence of cohort participation on decision making for early-phase clinical trials recruited from within the cohort, and the impact of the relationships that exist between researchers and participants. These data support the concept of cohorts as communities of persons, where identity is shaped, in part, through cohort experiences. The social meanings associated with cohorts have implications for the ethics of cohort-based research, as social contexts inevitably affect the ways that ethical concerns manifest.

## Introduction

Research cohorts study morbidity and mortality longitudinally among individuals generally identified by birth year, other demographic traits or as living with or at risk for specific disease conditions. In many research cohorts these members are requested to donate biospecimens and personal and medical information. Over time, new members may join the research cohort and new studies may recruit from the larger cohort.

Among the best-known research cohorts are the Whitehall cohort studies of British civil servants. Monitored over decades in the 20th century, the two Whitehall cohorts provided seminal data on the relationship between social status and mortality.^1^ In the USA, a similar example is the National Longitudinal Study of Adolescent to Adult Health,^2^ where a nationally representative sample of US adolescents completed five waves of data collection about social, environmental and biological determinants of adolescent health. Another well-known US study is the Framingham Heart Study,^3^ which began in 1948 and followed a random sample of households in Framingham, Massachusetts, a predominantly white community, to assess factors related to cardiovascular disease; a similar community-based cohort was also established in Jackson, Mississippi,^4^ to investigate environmental and genetic factors associated with cardiovascular disease among African Americans. Several cohort studies feature qualitative data only, e.g., longitudinal qualitative cohorts such as the UK Quality of Life study,^5^ the longitudinal study of settlement and well-being of Australian refugees^6^ and a cohort of healthy phase 1 volunteers in the USA.^7^ Qualitative research may also be embedded within quantitative cohorts, as exemplified by the recent report of Marques colleagues^8^ on a multimethod study of participation and attrition in a European cohort study.

Research cohorts focus on health problems relevant to local populations. In low- and middle-income countries (LMICs), examples include studies on infant and child mortality and/or infectious diseases such as human immunodeficiency virus (HIV)/acquired immunodeficiency syndrome (AIDS), cholera, malaria and tuberculosis. One such study is the 1993 Pelotas (Brazil) Birth Cohort Study, which built upon a 1982 cohort of infants and focuses on social and biomedical predictors of infant and child health over time. It is funded by the European Economic Commission in collaboration with the London School of Hygiene and Tropical Medicine and the Escuela Andaluza de Salud Publica from Granada.^9^

In this article we present new perspectives on the meaning and importance of research cohorts and their ethical implications. We first summarize traditional definitions of scientific value in cohort designs and challenges that may arise. We then reflect on cohorts as social communities, drawing from the anthropological and bioethics literature, including our earlier work on research relationships^10^ and our ongoing study of a research cohort in Thailand.^11^^,^^12^ Next, using published data from our research on decision making for Thai cohort members invited to participate in additional trials, we illustrate the meanings of cohort membership in one particular sociocultural context. These, in turn, have implications for the ways that ethical concerns emerge. We conclude with suggested areas of additional scholarship.

### Cohorts as collections of bodies

#### How are research cohorts defined?

Research cohorts are groups of volunteer participants from whom data are collected, stored and analysed over time to address biomedical as well as sociological questions. Their longitudinal designs help investigators understand complex disease aetiology, providing fundamental insights into environmental, lifestyle, clinical and genetic determinants of disease and its outcomes.

#### What value do cohorts offer?

Biomedical research cohorts, which tend to be expensive to develop and maintain, are typically justified by two types of value to investigators. First, cohort studies provide scientific value as a method to explore causal relationships about population- and individual-level predictors of health and disease that are not possible with one-time cross-sectional study designs, which only produce correlational data. Second, cohort studies provide an efficiency value for subsequent interventional research; that is, once established, cohorts provide a less costly route to locate and recruit participants for subsequent studies. This advantage has given rise to the practice of recruiting for and conducting ‘trials within cohorts,’ increasingly using randomized clinical trial designs. One such design—the cohort multiple randomized controlled trial, as described by Reeves et al.^13^—employs novel intervention trials efficiently within large longitudinal cohorts of patients, resulting in trial procedures more closely aligned to standard practice. Here we advance a potential third value, the benefits to participants from being part of a research cohort, including a trial conducted within that cohort, which contributes to knowledge and future treatments as well as improved medical care and ancillary medical care provision.

#### What scientific challenges may arise?

Despite the value of cohort-based research, there are certain scientific drawbacks. For example, one of the primary rationales for the creation of a cohort—to measure causal relationships over time—may be threatened by disturbances in the environment and individual behaviours caused by the cohort's existence. These potential disturbances must always be considered when attempting to generalize findings from cohort research to the populations from which they were drawn. Recruiting for trials within cohorts also further exacerbates concerns about the generalization of cohort data. Although recruiting trials from cohorts is pragmatic, there are associated methodological limitations debated within the literature, such as low statistical power and internal validity biases.^13^^,^^14^ Furthermore, initiating trials within cohorts may be confusing to cohort members, necessitating clear boundaries between cohort membership and trial participation.^14^^,^^15^ Lastly, as cohorts change over time, including their size, analytical design, environment and funding, so too does the feasibility of answering the originating and emerging scientific questions.

Like all studies, research cohorts require attention to informed consent and governance for collection of a range of specimens and types of data, including clinical records, genetic data and data on behaviours, environment and the community. Furthermore, as studies that take place over time, governance procedures may shift in response to internal or external forces. Expectations of the responsibilities and potential benefits associated with cohort membership may change. For example, norms about obligations to return research results may shift (e.g., Baggaley et al.^16^ on changing norms for returning HIV results), as may procedures for informing cohort members about advances in scientific knowledge, addressing benefits to the community and sharing access to research data with other investigators. In addition, when cohort sites are dispersed globally, and governance rules and standards of care vary by country, the practicalities of addressing these governance concerns becomes more difficult. Finally, challenges for studies recruiting in resource-constrained settings may be magnified in the case of longitudinal research cohorts. Harpham et al.^17^ discuss these challenges: how recruiting cohorts from clinics and hospitals can bias samples towards wealthier participants in low-income countries; difficulties in tracking individuals over time, especially when they move out of catchment areas and/or there is limited access to communication technology; and challenges in finding continuous material and human resource support for data storage and management in studies that may take decades. They conclude, ‘[these] studies require long term funding, a stable institution and an acceptance that there will be limited value for money in terms of results from early stages, with greater benefits accumulating in the study's mature years’.^17^

#### What ethical challenges may arise?

When ethical concerns about cohort studies are raised, they often focus on potential exploitation of individuals in the cohort or the communities where cohort members live. These concerns include how research subjects are ethically identified, recruited and retained; whether certain groups are neglected or unfairly targeted; whether the research conducted has the potential to benefit or adversely impact the community of interest; what obligations investigators have, including whether to return study results; and whether and how investigators experience and address conflicts of interest.

Many ethical issues are exacerbated when cohorts are recruited in LMICs, including the potential to receive medical care (often temporarily) that is superior to what is otherwise available,^18^ access to medical expertise unavailable in other settings and reimbursements that may be attractive even to the extent of being coercive for certain participants.^19^ Furthermore, clinical research in LMICs may test experimental interventions that are not guaranteed to be available or affordable after the trial is over.

Some analyses address broader structural questions about how cohorts are situated. For example, Carrel and Rennie^20^ discuss ethical considerations in longitudinal surveillance cohorts in Asia and sub-Saharan Africa, observing that ‘the indistinct positions of such surveillance systems, often inhabiting an area between research, treatment and population health monitoring, means that the necessity of and responsibility for ethical oversight (including the obligation to obtain informed consent) is unclear’. Wherever cohort studies are carried out, ethical considerations are raised by dependence on funding agencies and organizations, relationships with external institutions or investigators and other socio-economic and political forces.

### Beyond bodies: cohorts as communities of persons

We propose that cohorts are not only collections of bodies but are also communities of persons whose membership expands in significance across time and place. Cohorts create value and social identities for members, investigators and organizations. Borrowing the concept of ‘imagined communities’ from Anderson,^21^ we argue that for investigators as well as participants, cohorts are not defined solely by biomedical objectives (e.g., principal investigator for a trial, inclusion/exclusion criteria for recruiting participants) but also are, to a greater or lesser extent, constructs of meaning. Meaning and perceived value are constructed by participants and investigators, continue over time and can lead to the development of a cohort identity. Embedded in the social meanings of cohorts are features with implications for the ethics of cohort-based research, as social contexts inevitably affect the ways that ethical concerns manifest. Our argument is based first on examples drawn from the literature and second from our research with members of an HIV research cohort in Bangkok.^11^^,^^12^

### Examples of cohort meaning and identity

Research cohorts create varying meanings for members and for investigators. Some research cohorts become communities for members after the cohorts are built, creating membership through the act of diagnosis or identification that leads to cohort recruitment. Other research cohorts build on pre-existing communities, often linked to a geographic location (e.g., the Pelotas Birth Cohort Study^9^). Social scientists Lappé and Jeffries Hein's^22^ research on placentas used in birth cohort studies is a rare instance of ethnographic work on a longitudinal research cohort, which indicates how scientific goals and procedures are entangled with participant–investigator relationships and the environment.

While a research cohort identity is just one of many identities that members hold,^23^ this identity may be important to members. This may be especially relevant when there is overlap with other established identities, such as disease communities, or as part of a famous study with well-known contributions to medical knowledge (e.g., the Framingham Heart Study). As a third-generation participant in the decades-long Framingham Heart Study said, ‘I'm happy that I can take part in it. It's done so much to aid major breakthroughs’.^24^ For other cohort members who are recruited on the basis of a sensitive or stigmatized behaviour or condition, such as sexually transmitted infections, genetic conditions or mental illness, cohort identity may not be as straightforward. For these members, the potential risk of being identified as a participant may outweigh or complicate the value of group identity; this may be especially true in countries where stigmatized behaviours are illegal.

For both cohort members and investigators, being part of a cohort may involve long-term, rewarding relationships between members and research staff that contribute to the cohort identify and meaning. These relationships may be deepened and complicated when clinical as well as non-clinical (e.g., epidemiological) research activities are involved. The work itself fosters diverse relationships, which over time may take on the features and governance of complex organizations, depending on size, longevity and the institutions to which they are responsible. For investigators, their own identities and investment in the research enterprise may become intertwined with the cohort; indeed, a successful cohort can make researchers’ careers and be part of how they are known.

In addition, the objectives that motivated the original establishment of the cohort may change, expand or decrease as scientific outcomes are produced, and these changes may impact the cohort's meaning, identity and expectations. Understanding this kind of evolution requires an appreciation of the momentum that comes with an existing cohort and obligations that may not have existed when it began. An example of this phenomenon is offered by Bandewar et al.,^23^ who describe the creation of a sex worker observational cohort in Majengo, Kenya, that has existed for over 30 years. It was established to reduce the barriers to healthcare, particularly for HIV and other sexually transmitted infections, yet cohort members gradually came to expect that investigators would address not only medical care, but provide a path out of sex work. Challenges to researcher roles exemplified by this case are discussed by Lavery et al.^25^ One interpretation is that the investigators tried to maintain the biomedical goals of the cohort by offering healthcare to participants; the participants, in requesting help with liberation from sex work, saw themselves and their relationship with the research team quite differently and wanted more than the original research offered. According to Tukai,^26^ over time the Majengo sex workers gained benefits such as education/empowerment in regard to decision making in research and advocacy for sex worker rights. The case illustrates evolving expectations on the part of both researchers and cohort members, which can create benefits and challenges for all involved.

### The South East Asia Research Collaboration in HIV  Study (SEARCH010/RV254) cohort case

To illustrate how cohorts are communities of persons, with their own meaning, identity and relationships, we describe the Thai SEARCH010/RV254 research cohort and our research with cohort researchers and members.

SEARCH010/RV254 is a longitudinal, observational research cohort of individuals identified and referred during acute HIV infection. Once individuals joined the cohort, they were immediately placed on antiretroviral treatment (ART) and followed up with regular HIV care.^27^ SEARCH010/RV254 was established in 2009 by the US Military HIV Research Program (MHRP) (clinicaltrials.gov, NCT00796146) in collaboration with the Thai Red Cross AIDS Research Centre.^28^ From the beginning, SEARCH010/RV254 investigators identified individuals with state-of-the-art testing across Bangkok, administered ART and followed them closely over time until their virus was suppressed to a level of <20 copies/mL. SEARCH010/RV254 investigators engaged the cohort to study the basic biology of early infection, disease incidence, viral diversity, host genetics and treatment outcomes. Protocols brought cohort members into the clinic every 6 months, at a minimum, for clinical evaluations and periodic specimen collections. Six optional procedures were also requested (genital secretions, lumbar puncture, magnetic resonance imaging, leucopheresis and colon and lymph node biopsies). By 2020, 643 individuals had been enrolled in the cohort, primarily young men (median age 25–28 y) who have sex with men. Worldwide, individuals identified at the early stages of HIV infection have become a priority for cutting-edge HIV remission (‘cure’) trials.^29^^,^^30^

The SEARCH010/RV254 cohort was established after ART was free for all in Thailand in 2006. Those enrolled were offered ART initiation no matter what their CD4 count. After 2014, ART was recommended in Thailand regardless of CD4 count, however, in most hospitals it still took weeks to months before it was initiated, compared with a few days in SEARCH010/RV254 patients. With same-day ART service integrated^31^ into the Thai Red Cross Anonymous Clinic in July 2017, the timing of ART initiation through routine service became quite similar to that of SEARCH010/RV254.

#### Remission (‘cure’) trials within the SEARCH010/RV254 cohort

Beginning in 2015, participants in four early phase remission trials were recruited from the cohort and the trials have been completed. Their aim is to understand factors related to long-term control of HIV. All the trials include analytic treatment interruption (ATI), a controversial procedure to test the efficacy of an intervention used to suppress HIV viral load by taking participants off of ART.^30^^,^^32^^–^^35^ Those undergoing ATI are followed in a controlled setting until viral rebound occurs, after which treatment is restarted.

#### Studying SEARCH010/RV254 cohort members invited to remission ‘cure’ trials within the cohort

Because SEARCH010/RV254 investigators were concerned about voluntary and informed consent for these controversial trials, our group was invited to study how cohort members decide to join or decline.^11^^,^^12^^,^^36^ This invitation forecast the importance to investigators of ethical treatment of cohort members as they embarked together on potentially risky trials within the cohort. Such investigator concern for cohort members is also seen in a recent study by Dahlin-Ivanoff et al.^37^ In 2016, with the support of the National Institutes of Health, collaborators from the MHRP and co-investigators from the USA and SEARCH010/RV254, we began to interview cohort members about decision making for these trials conducted within the cohort. For each remission trial participant (n=54) and decliner (n=20), we asked about the experience of HIV diagnosis and rapid recruitment to SEARCH010/RV254, where they received immediate ART initiation.

Cohort members recruited to remission trials reported that being part of the SEARCH010/RV254 research cohort was a motivator. They wanted to help the research team with their scientific objectives and they trusted that the staff would keep them safe. As we noted in a prior publication,^11^ ‘Some focused on helping the SEARCH/RV254 team or the cohort. Many participants wanted to aid the research team who “work so hard and always encourage [us]…” [while another noted], “Now I have my brothers and sisters and we talk and encourage each other.”’ In our study examining individual decisions to join or decline participation in the SEARCH010/RV254 cohort, decliners described similar relationships, and for some, regret that they decided against participating.^11^^,^^12^ For some individuals who joined the cohort, meeting the inclusion/exclusion criteria reinforced a belief that they were specially qualified, and for a few, perhaps also obligated to participate in the remission trials. As our interviews revealed, ‘very early HIV diagnosis created a sense of having “special bodies” for research that offered the potential for reciprocal benefit to self and others’.^12^ Some saw participation in ‘cure’ trials as the opportunity to test or ‘challenge’ their bodies for science, and when their bodies ‘failed’ (e.g., viral rebound), this might be internalized as a personal defeat. These views of being special were bolstered by the trial team in their descriptions of the SEARCH010/RV254 cohort and the justifications for conducting the remission trials.

From the interviews we also learned about the importance of SEARCH010/RV254 in helping participants cope with their HIV diagnoses. Cohort members describe the close, ongoing relationships they have developed with nurses, study coordinators and physician-investigators.^11^^,^^12^ This may be particularly important in light of the high HIV stigma in Thai society^38^ and the limited disclosure of HIV status by cohort members: approximately 25% of cohort members in a survey substudy reported they had never disclosed their HIV status to anyone beyond SEARCH010/RV254 staff and another 20% only disclosed to one family member or friend.^39^

Interviews revealed a sense of newly found community not based on gatherings of individuals with HIV or support groups outside the clinic visits, as such meetings were seen by many as potential risks of revealing their HIV status. In fact, protecting the confidentiality of research participation was highly valued by both cohort members and investigators. Rather, this sense of community was based on trusting relationships and reciprocity with the research teams and the institution and based on a pervasive optimism about scientific advancement. In sum, being part of the SEARCH010/RV254 research cohort seemed to occupy a central identity for many cohort members in adapting to their HIV-positive status, exemplifying the personal meaning and positive psychological aspects of cohort membership.

## Discussion

In this article we explore key features of cohorts developed for research purposes and their scientific, practical and ethical challenges. We summarize the literature to provide context and describe the SEARCH010/RV254 HIV research cohort in Bangkok as an illustrative case. While our preliminary review of biomedical cohorts and the social meanings of cohorts is far from exhaustive, we elucidate two ways of understanding cohorts in research. The first is the traditional biomedical conception of cohorts as collections of bodies, which offer significant scientific value as well as associated problems and governance challenges. The second defines cohorts as communities of persons, creating social identities for both investigators and cohort members. As cohorts exist over time, these meanings co-mingle in important ways that illuminate some of the scientific, practical and ethical dilemmas that can arise.

Important questions arise from understanding cohorts as communities of persons that should be the subject of future scholarship. For example, what expectations may arise regarding benefits, risks and privacy when participants perceive themselves to be part of a supportive community? How might this understanding impact retention rates? When participants are recruited to additional studies in the cohort, do they feel an obligation to agree based on their understanding of the cohort as a community? How might this affect investigator–participant relationships in positive and more problematic ways, and the feelings of reciprocity between them?

The SEARCH010/RV254 case provides some answers to these questions. The SEARCH010/RV254 cohort identity comprises both a biomedical understanding of the meaning of HIV and diagnosis in the acute phase, including the potential medical benefits of joining the cohort, and a sense of specialness and community due to the shared acute identity with other cohort members. We also observed evidence of cohorts as communities of persons from SEARCH010/RV254 investigators, with whom we have collaborated for over four years. We observed that they have a strong identity as investigators and caregivers for the cohort, including a clear sense of the positives and negatives for participants of being part of the cohort. Based on biomedical evidence,^40^ they believe that early diagnosis and treatment conveys a medical benefit—part of their recruitment materials for the cohort. They are invested personally and professionally and actively encourage this identification.

The offer of ‘cure’ trials within the SEARCH010/RV254 cohort is another context in which we found evidence of ‘cohort as community.’ From the perspective of investigators, trials within cohorts can fully benefit from the expensive commodity of the cohort and the promise of scientific advances. Remission trials often seek individuals diagnosed at the very early stage of infection, making this cohort particularly advantageous from a scientific perspective. At the individual level, investigators may be concerned about possible risks of participation, yet in advancing science, they also offer cohort members the opportunity for access to experimental treatments unavailable to others, possibly benefiting the participant. As mentioned above, the initial invitation to conduct our decision-making study was based on the investigators’ concern that SEARCH010/RV254 might unintentionally take advantage of participants’ positive sense of cohort identity to recruit people into greater than minimal risk studies. In a recent article about our research collaboration with SEARCH010/RV254, co-authored by members of both teams, we address reciprocity by defining rare cases of potential coercion when confidential interview data should be shared.^36^

Cohort members’ responses about ‘cure’ trials reflect concerns about voluntariness that seem to arise from their experience of the cohort as a community: feelings of reciprocity or obligation to participate seem linked to relationships with investigators, trust that they will be cared for by the team and a sense of community with other cohort members; for the large majority we found evidence of valid informed consent, regardless of this reciprocity.^11^^,^^12^ Because SEARCH010/RV254 encompasses both clinical and research relationships, their multidimensional relationships generate ethical and caregiving obligations. This is not uncommon in clinical research but requires transparency and anticipatory attention. Lastly, our data suggest that because cohort members see ‘cure’ trials as ways of finding out what their bodies are capable of, they were incorporating, to a small degree, the biomedical ‘collection of bodies’ paradigm of SEARCH010/RV254 membership into what was also participation in a meaningful project.

It is important to acknowledge that material presented or referenced in this article are descriptive and highly context-specific. Our findings are not generalizable, but rather are meant to illustrate the need to study underrecognized features of research cohorts. In the SEARCH010/RV254 cohort, for example, a key differentiating feature is their internalized stigma and lack of disclosure of HIV status,^39^ which in turn seemed to generate particularly close relationships with the clinical and research staff. Many other cohorts retain value because they allow interparticipant engagement and a public identity, e.g., ‘I'm part of the Framingham cohort’. Although these interparticipant relationships are infrequent in SEARCH010/RV254, we still find evidence of a cohort identity that is semiprivate—e.g., important in giving meaning to participants’ lived experience with HIV, which is reinforced in their interactions with the SEARCH010/RV254 investigators. The processes by which these relationships have emerged are particular to this case, but cohort relationships and identities themselves likely permeate this important research entity.

## Conclusions

Moving forward, scholarship on key ethical implications of research cohorts—for both investigators and cohort members—should address factors related to their generation, persistence and evolution over time. Specifically, a ‘responsible conduct of cohort research’ framework could address specific considerations that arise when cohorts are established and longitudinal research is carried out. As well, attention should turn to ethical recruitment for cohort-based clinical trials and ethical considerations when bringing a research cohort to an end. Case-based explorations of research cohorts will reveal parameters that should be important for ethical assessments. Cohort investigators might consider their evolving identities as cohort leaders, whether initiating a cohort or managing changing expectations and responsibilities over time. Potential collaborations with social and behavioural researchers might further define these issues and address them.^36^ More integrative, generalizable conclusions await these kinds of studies.
